# A Systematic Review of the Effectiveness of Acceptance and Commitment Therapy (ACT) for Body Image Dissatisfaction and Weight Self-Stigma in Adults

**DOI:** 10.1007/s10879-018-9384-0

**Published:** 2018-02-21

**Authors:** Catrin Griffiths, Heidi Williamson, Fabio Zucchelli, Nicole Paraskeva, Tim Moss

**Affiliations:** Centre for Appearance Research, University of the West of England (UWE), Frenchay Campus, Coldharbour Lane, Bristol, BS16 1QY UK

**Keywords:** Acceptance and Commitment Therapy, ACT, Body image, Weight self-stigma, Adults

## Abstract

Body image dissatisfaction (BID) and weight self-stigma are prevalent and associated with physical and psychological ill-health. Acceptance and Commitment Therapy (ACT) is increasingly employed for both, yet little is known about its effectiveness. Searches of 12 databases identified six studies using online, face-to-face or self-help ACT interventions for BID or weight self-stigma, of varying duration and intensity. Their effectiveness and quality were evaluated. Two reported improved BID, three improved weight self-stigma, and one reported no impact on weight self-stigma. Methodological issues (small sample sizes, lack of allocation concealment, attention control and long-term follow up) impacted the validity of findings. Due to the small number of studies and poor study quality, the effectiveness of ACT for BID and weight self-stigma remains unclear. Nonetheless findings suggest psychological flexibility may facilitate reduction in BID and weight self-stigma and indicate that brief online as well as lengthy face-to-face delivery may be useful. Suggestions for further research are made.

## Introduction

Around 61–93% of individuals report body image dissatisfaction (BID) (Diedrichs et al. in preparation; Liossi 2003). Growing recognition of the worldwide prevalence of BID and its negative impact on psychological and physical well-being has instigated demands for its recognition as a major public health concern (Bucchianeri and Newmark-Sztainer 2014). Body image, defined as “one’s perceptions and attitudes in relation to one’s own physical characteristics” (Cash and Fleming 2002, p. 455) such as weight, shape, height and skin colour, is a multi-dimensional construct that incorporates cognitive, affective, behavioural and perceptual facets. Negative body image, generally assessed using self-report measures of BID, is defined as “negative subjective evaluations of one’s physical body” (Stice and Shaw 2002, p. 985) and can include negative appearance-related thoughts (e.g., ‘I am ugly’), feelings (e.g., self-consciousness, appearance-anxiety) and behaviours (e.g. excessive appearance-checking). Those experiencing positive body image, often assessed using self-report measures of body appreciation, tend to respect and appreciate their body regardless of its appearance, engage in body-protective health behaviours and reject “unrealistic media appearance ideals” (Tylka and Homan 2015, p. 91).

BID can span a lifetime, and disproportionately affects women, although men are increasingly affected (Tiggemann 2004). BID is associated with heightened anxiety, depression, low self-esteem, reduced quality of life and risky health behaviours including disordered eating. Those affected may use crash-diets, laxative and diet pills and vomiting in attempt to change their body shape and weight (Bucchianeri and Newmark-Sztainer 2014; Ganem and Morera 2009). Identifying effective psychological interventions to reduce BID is therefore a priority for healthcare services.

Despite extreme and widespread social pressure to be thin in society, rates of obesity have substantially increased over the past 50 years (Finucane et al. 2011). Recent figures show 40.4% of women and 35% of men in the US are classed as obese and this is set to increase to 50% by 2030 (Wang et al. 2011). Consequently, the gap between the ‘ideal’ body size and the body of an overweight or obese person is growing. As the discrepancy between ‘ideal’ and actual body size increases, so does the risk of BID and weight-related self-stigma (Bessenoff and Snow 2006).

Overweight or obese people are commonly subjected to pervasive weight-related social discrimination and are often stereotyped as unattractive, lazy, immoral and dishonest (Latner et al. 2005). Individuals can internalise this stigma by accepting these beliefs, fearing stigma from others, engaging in self-devaluation, and holding weight-related self-stigmatising attitudes. This is collectively known as weight self-stigma (Lillis et al. 2010). Weight self-stigma is closely related to BID, and has been associated with a range of negative outcomes including feelings of isolation, poor psychological functioning, binge eating, depression and other psychiatric symptoms (Wott and Carels 2010). Rather than serving as a motivator for weight loss, those experiencing weight self-stigma are more likely to engage in unhealthy behaviours that impede weight loss. For example, Carels et al. (2009) identified that baseline levels of weight self-stigma predicted poorer self-monitoring, lower energy expenditure and greater calorie intake in a sample of overweight and obese adults participating in a weight loss programme. Thus it is vital for health interventions to target the psychological processes that influence weight gain, to help individuals become more accepting of their weight and eating-related experiences, and develop healthier behaviours and promote quality of life (Palmeira et al. 2017a).

Given the potential psychological difficulties associated with BID and weight self-stigma, and the role these can play in instigating and perpetuating disordered eating and obesity, it is vital to identify effective psychological interventions that ameliorate BID and weight self-stigma. Currently very little research has evaluated psychological interventions to reduce weight self-stigma. In contrast a range of psychosocial approaches have been evaluated that target BID. These are broadly grouped into interventions that use: cognitive behavioural therapy (CBT) to change negative or unpleasant thoughts, feelings and behaviours that influence negative body image; media literacy techniques to enable participants to critically evaluate and challenge media images and messages that promote female and male ‘ideal body images’ (i.e. the thin and muscular ideal); and psychoeducation, which aims to increase awareness of the factors that influence body image and knowledge of the impact negative body image has on psychological and physical well-being (Alleva et al. 2015). However, a meta-analytic review of 62 randomised controlled trials of body image interventions (which included CBT, media literacy and psychoeducation) found interventions engendered only small improvements in body image (Alleva et al. 2015). The authors acknowledged the need for large-scale, high-quality trials in this area and highlighted that relatively new interventions, such as mindfulness-based interventions, require empirical attention. Acceptance and Commitment Therapy (ACT; Hayes et al. 1999) is one such mindfulness-based intervention.

The rationale for using ACT in this context is based on empirical research that suggests psychological inflexibility (i.e. the tendency to rigidly attempt to control or avoid difficult internal experiences and to place extreme importance on the literal content of thoughts and feelings) has a significant relationship with both BID and weight self-stigma (Lillis et al. 2011). For example, Mancuso (2016) reported that body image inflexibility mediated the relationship between BID and both experiential avoidance and appearance-fixing in 156 women, meaning those with greater body image flexibility were less likely to employ these maladaptive coping strategies. Similarly Webb (2015) identified that older adolescent girls with greater body image psychological inflexibility were less likely to engage in body appreciation behaviours, and that this held when controlling for body mass index (BMI). In an obese sample, psychological flexibility and weight self-stigma was found to significantly predict health-related quality of life (Lillis et al. 2011). Lillis et al. also found that both psychological flexibility and weight self-stigma worked in combination (and independently) to fully mediate the relationship between BMI and health-related quality of life. These findings suggest that an increase in psychological flexibility relating to one’s body image/weight (i.e. being aware of, allowing and accepting difficult body image experiences) may reduce BID and weight self-stigma.

ACT aims to increase an individual’s psychological flexibility in how they experience difficult thoughts and feelings about their physical appearance via six core processes of change collectively known as the “ACT Hexaflex”: acceptance, cognitive defusion, contact with the present moment, self-as-context, value-driven behaviour, and commitment to value-driven behaviours (Hayes et al. 2006). ACT theory holds that the content of a ‘dysfunctional’ thought (e.g. “I’m fat”) is not inherently problematic and in need of modification. Rather, it is psychological inflexibility—identification with thoughts as self-evident facts (*cognitive fusion*) and avoidance of their associated aversive affective state (i.e. *experiential avoidance*)—that predicts negative affective and behavioural outcomes. ACT teaches mindfulness techniques and commitment to one’s values to increase psychological flexibility. Webb et al. (2014) suggest that encouraging body image flexibility via mindful awareness and acceptance towards such thoughts, and using values to guide one’s response (e.g. having negative body image thoughts but continuing to exercise at the gym by being guided by a value to stay healthy), will produce more adaptive weight loss behaviour and ultimately improve body image.

ACT can also enhance emotional well-being relevant to BID and weight self-stigma via the cultivation of a non-critical self-image as a result of non-judgemental self-awareness (Kristeller et al. 2006), which may be especially beneficial for those who negatively evaluate their bodies. ACT may also improve BID and weight self-stigma by encouraging exposure to difficult thoughts and emotions, leading to a desensitisation to difficult appearance-related thoughts and feelings and a subsequent reduction in distress. Lastly, developing acceptance may increase levels of meta-cognitive awareness of one’s thought processes. This increases the likelihood of thoughts and feelings being simply observed, increasing a sense of self-control, which can reduce automatic reactions (Segal et al. 2002).

Currently little is known about the overall effectiveness of ACT for BID or weight self-stigma. However, ACT has been found to be effective at reducing self-stigma in other contexts where social discrimination is common, including among those with substance abuse (Luoma et al. 2012), sexual orientation difficulties (Yadavaia and Hayes 2012) and HIV (Skinta et al. 2014). Work exploring the impact of ACT in the highly related area of clinically diagnosed eating disorders is also still in its infancy. Manlick et al. (2012) literature review summarised empirical research illustrating that ACT is widely used and tested among those diagnosed with an eating disorder, and cited findings related to the impact of ACT on body image among this population. However, the authors did not evaluate the scientific quality of the findings. No systematic review to date has critically evaluated the effectiveness of research that has investigated ACT for BID or weight self-stigma. This is surprising given the widespread prevalence of both BID (61–93%) and obesity (35–40.4%) compared to rates of diagnosed eating disorders such as anorexia (5%), bulimia (1–3%) and binge eating disorder (1–3%), and the increasing evidence of the predictive role BID and weight self-stigma play in these conditions. It also surprising given the growth of ACT within the field of clinically diagnosed eating disorders and body image. The aim of this systematic review was therefore to identify studies testing ACT interventions for BID and/or weight self-stigma in adults without a clinical eating disorder, evaluate the studies’ quality, and synthesise the findings.

## Method

This review used the PRISMA checklist for reporting systematic reviews (Moher et al. 2009) and guidance published by the Cochrane Collaboration (Higgins and Green 2011). No protocol for this systematic review is available publicly but further information can be provided by contacting the first author.

### Inclusion and Exclusion Criteria

Studies were eligible if they included adult participants (over 18 years old) who had received an ACT intervention, whether individually or in a group, via single or multiple sessions, provided by a clinician or researcher in a hospital, community or university setting. Studies with and without a control were included to ensure the search was inclusive of all relevant literature. Studies had to include a quantitative outcome measure that assessed BID or weight self-stigma (either post-intervention or longer follow-up). Studies had to be published in English and could be published or unpublished. Studies targeting participants diagnosed with eating disorders, namely anorexia nervosa, bulimia nervosa or binge eating disorder, were excluded.

### Search Strategy

**Fig. 1:**
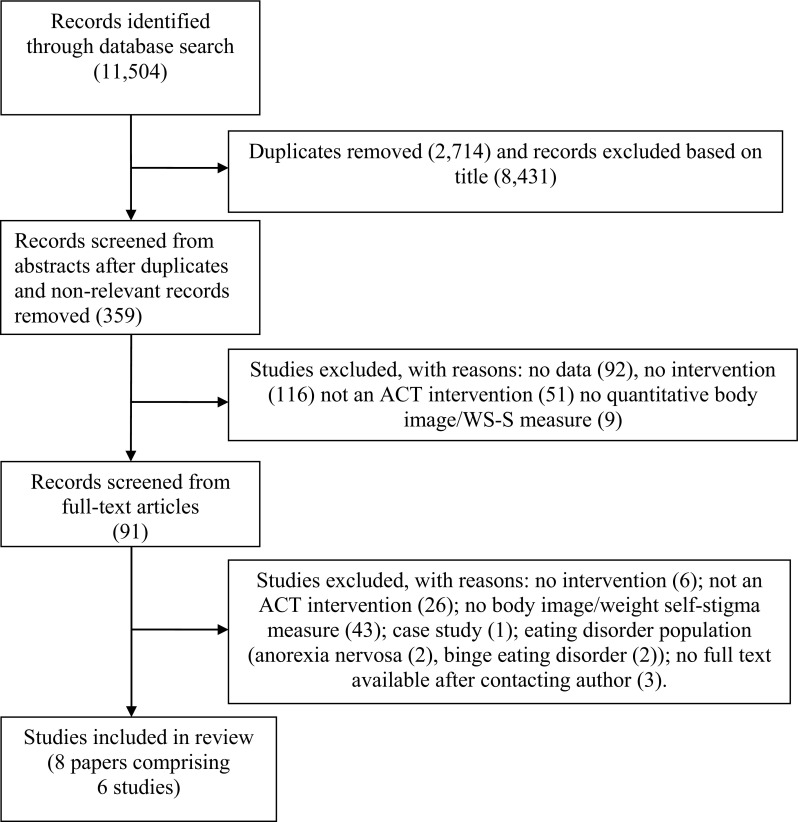
Flow diagram of systematic selection of papers in review

A literature search on ACT for improving BID and/or weight self-stigma was conducted utilising the electronic databases Amed, Cinahl Plus, Medline, Psycarticles, PsycINFO, Web of Science, the Cochrane Library, Assia, British Humanities Index, IBSS, PILOTS and Social Services Abstracts. The search terms were (“acceptance” OR “defusion”) AND (“body” OR “appearance” OR “weight” OR “shape” OR “eating” OR “visible difference*” OR “disfigure*” OR “overweight” OR “obesity” OR “obese”). The authors checked the search terms with two experts in the field of body image research to ensure for accuracy and comprehensiveness, resulting in the search term “disfigure*” being added. The terms were searched for within the title and abstract of articles. No starting time period was specified but records were searched up until October 2017. Forward and backward snowballing techniques were also applied (via Web of Science cited reference searches and reading reference lists from key papers), and a search of unpublished and grey papers was conducted to mitigate the effects of publication bias (Song et al. 2010). Unpublished or missing information was requested from the study’s corresponding author when necessary. Two reviewers independently extracted data, and any discrepancies were discussed between reviewers and resolved through consensus. Figure 1 shows the study search results and selection process.

### Assessment of Risk of Bias

**Fig. 2:**
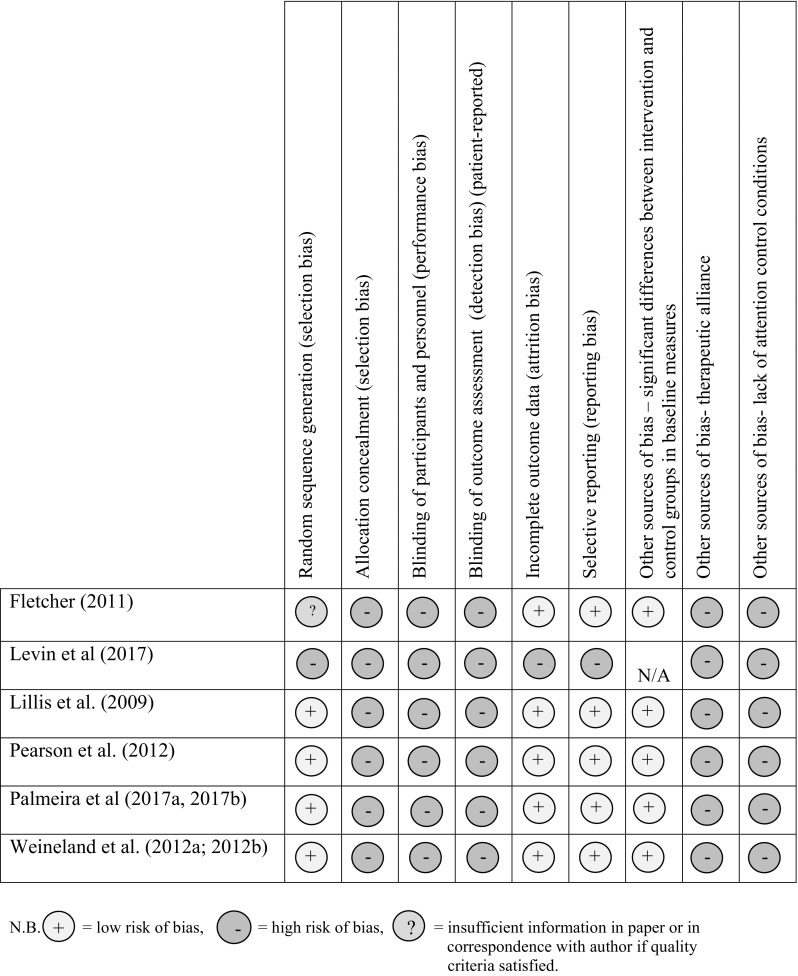
The risk of bias of individual studies using the Cochrane risk of bias tool. *N.B*. low risk of bias, = high risk of bias, = insufficient information in paper or in correspondence with author if quality criteria satisfied

The methodological quality of each study was assessed by two reviewers according to the recommendations of the Cochrane Collaboration using the Cochrane risk of bias tool (Higgins and Green 2011). This tool assesses the generation of allocation sequence, allocation concealment, blinding of outcome assessors, completeness of follow-up data, selective reporting, and other sources of bias (see Fig. 2).

## Results

### Study Characteristics

The six individual studies in the review incorporated 351 individual participants (326 females and 25 males) and were published between 2009 and 2017 (Table 1). All were published in peer-reviewed journals apart from Fletcher’s (2011) study, which was a doctorate thesis. The ethnicity of the participants was reported in three out of six studies (Fletcher 2011; Lillis et al. 2009; Levin et al. 2017) and the majority ethnic group was Caucasian. The average BMI of participants across the studies was 33.20 (range 22.38–38.01), which falls at the low end of the obese category. Four studies were conducted in the US (Fletcher 2011; Levin et al. 2017; Lillis et al. 2009; Pearson et al. 2012), Portugal (Palmeira et al. 2017a, b) and Sweden (Weineland et al. 2012a). Two studies included participants who had previously attended a weight-loss program (Fletcher 2011; Lillis et al. 2009); one recruited participants who self-reported weight self-stigma and who had previously participated in a weight loss program (Levin et al. 2017); one recruited participants actively enrolled in a weight loss program (Palmeira et al. 2017a, b); one recruited participants who had undergone bariatric surgery (Weineland et al. 2012a); and one enrolled women who self-reported BID (Pearson et al. 2012).

**Table 1:** Data extraction table of included studies

Author	Participants	Details of intervention	Control	Primary outcome measures	Main findings
Fletcher (2011)	T: 72, I: 36, C: 36 *Population type*: Current or past patients of structured weight-loss programme *Gender*: 60 female, 12 male *Age*: 40–64 years, M = 52.6 (no SD reported) *Ethnicity*: 64 Caucasian, 5 Hispanic/Latino, 2 African American, 1 unknown *BMI: M* = 35.45 (no SD reported)	*Setting*: University laboratory hosting structured weight-loss programme *Intervention*: 1 × 6 h ACT workshop to increase physical activity: introduced ACT concepts covered barriers to physical activity, focused on weight stigma. Facilitated by 3 × Doctoral level graduate students with > 2 years’ experience of delivering ACT	Waitlist control (completed workshop after 3 month follow-up)	*Outcome measure*: 30-item Weight Self-Stigma Questionnaire (WSSQ) (Lillis et al.; 2009). No process measures *Timing of administration* Pre- and directly post-workshop, 3 month follow-up	No significant differences in WSQ scores between the ACT and waitlist control conditions at post-workshop assessment or 3-month follow-up *Attrition*: 59 of 72 dropped out (18.9%). ACT group: 5 did not attend workshop, 2 dropped out after workshop attendance, 29 of 36 (80.6%) completed all 3 assessments. Control group: 1 dropped before completing any assessments, 2 dropped out after first assessment, 30 of 36 (83.3%) completed all 3 assessments
Levin et al. (2017)	T: 13, I: 13, No control *Population type*: Individuals self-reporting weight self-stigma with a BMI > 27.5 *Gender*: 12 female and 1 male *Age*: 18–60, M = 35.10, SD = 12.63 *Ethnicity*: 9 Caucasian, 1 Asian American *BMI*: M = 34.11, SD = 5.21	*Setting*: Participant’s’ home *Intervention*: ACT self-guided workbook for weight concerns and weight self-stigma; 7 weekly sessions plus weekly 5–10 min coaching. Facilitated by 2 ACT-trained Clinical Psychology Doctoral students	No control	*Outcome measure*: 12-item Weight Self-Stigma Questionnaire (WSSQ; Lillis et al. 2010) Process measures: Acceptance and Action Questionnaire for Weight **(**AAQW; Lillis and Hayes 2008);Valuing Questionnaire (VQ, Smout et al. 2014) *Timing of administration*: pre- and directly post-intervention, 3-month follow up	*Compared to baseline*: ACT condition significantly lowered weight self-stigma at 3 month follow up with large effect size (M difference = 15.20, SE = 2.63, p < 0.001, Cohen’s d = 2.63) *3 month follow up*: ACT condition significantly increased weight-related psychological flexibility with large effect size (M difference = 42.70, SE = 5.77, p < 0.001, Cohen’s d = 2.41); significantly reduced obstruction with large effect size (i.e. how much barriers get in the way of valued action) (M difference = 9.95, SE = 1.16, p < 0.001, Cohen’s d = 2.80); significantly increased progress in valued action with large effect size (i.e. engaging in meaningful patterns of activity) (M difference = 7.23, SE = 1.41, p < 0.001, Cohen’s d = 1.41). However no mediation analysis was conducted on the process variables on weight self-stigma *Attrition*: 3 dropped out within 3 weeks of using the self-help book
Lillis et al. (2009)	T: 84, I: 44, C: 40 *Population type*: Completed at least 6 months of a weight loss program in the past 2 years *Gender*: 76 females and 8 males *Age*: 39–64.4, M = 50.75, (no SD reported) *Ethnicity*: 78 Caucasian, 4 Hispanic, 2 African American *BMI*: M = 33.04	*Setting*: university laboratory *Intervention*: 1 day (6 h) group ACT workshop to improve the quality of life of obese people. Focused on obesity stigma, increasing acceptance, mindfulness and values based action Facilitated by experienced ACT facilitator and ACT-trained clinical psychologist trainee	Waiting list control (completed workshop after 3 month follow-up)	*Outcome measure*: 30-item Weight Self-Stigma Questionnaire (WSSQ) (not validated). Designed for this study as no relevant measure of weight-related self-stigma existed *Process measures*: Acceptance and Action Questionnaire for Weight **(**AAQ-W; Lillis and Hayes 2008); Acceptance and Action Questionnaire (Bond and Bunce 2003) *Timing of administration*: pre-intervention, 3 months post-intervention	*3 month follow up*: compared to control ACT condition significantly lowered levels of weight-related self-stigma with large effect size (F (1, 83.00) = 24.34, p < 0.001, partial η^2^ = .23, d = 1.07); improved weight-related psychological flexibility (p = 0.00, CI 4.55–15.01) and general psychological flexibility (AAQ) (p < 0.01, CI 0.82–7.69); significantly mediated reductions in weight related self-stigma; resulted in greater weight loss (post analyses showed improvements in weight related self-stigma not due to weight loss in ACT group) *Attrition*: 3 in ACT condition completed assessment procedures but dropped out before attending workshop
Palmeira et al. (2017a, b)	T: 73, I: 36, C: 37 *Population type*: Overweight or obese women self reporting weight self-stigma *Gender*: all female Age: 18–55, M = 42.35, SD = 8.58 BMI: M = 34.24, SD = 5.05 Ethnicity: not reported	*Setting*: hospital *Intervention*: ACT and self-compassion 12 session group intervention designed to reduce weight self-stigma *Facilitator*: ACT-trained clinical psychologist and one clinical psychology masters student	*Treatment as Usual*: maintained medical and nutritional appointments	*Outcome measure*: 12-item Weight Self-Stigma Questionnaire (Portuguese Version) (WSSQ; Lillis et al. 2010; Palmeira et al. 2017a, b) *Process measures*: Acceptance and Action Questionnaire for Weight-Related Difficulties-Revised (AAQW-R, Palmeira, et al. 2016); Other as Shamer Scale (OAS; Goss et al. 1994; Matos et al. 2016); Self-Compassion Scale (SCS; Castilho et al. 2015; Neff 2003) (contains one positive and one negative subscale: self-compassion and self-judgement) Five Facet Mindfulness Questionnaire—15 (FFMQ-15, Baer et al. 2006) *Timing of administration*: Pre- and directly post-intervention or within 2-weeks post intervention, 3-month follow up	*Post intervention*: compared to control ACT condition significantly reduced weight self-stigma with large effect size (F(1, 57) 16.94, p < 0.001, η^2^ = 0.24).; Reductions in weight self-stigma were mediated by decreased levels of weight-related psychological inflexibility with large effect size (p < 0.01, d = 3.00), shame with large effect size (p < 0.001, d = 1.96,a), self-judgment patterns with large effect size (p < 0.01, d = 1.80) and increased self-compassion with large effect size (p < 0.05, d = 0.96) and mindfulness with large effect size (p < 0.001, d = 1.53) 3*-month follow* up: Reductions in weight self-stigma from baseline to post-treatment were maintained in the intervention group. However no comparison of the ACT intervention with the control group was included. ACT resulted in greater weight loss (BMI) compared to control. However post analyses showed that improvements in weight related self-stigma not due to weight loss in ACT group *Attrition*: 9 of 36 in ACT group and 5 of 37 in TAU group dropped out, 2 more dropped out by 3 month follow up
Pearson et al. (2012)	T: 73, I: 39, C: 34 *Population type*: Women with body dissatisfaction responding to advert *Gender*: all female *Age*: 18–68, M = 43.4, SD = 14.7 *BMI*: M = 29.3, SD = 6.0 *Ethnicity*: not reported	*Setting*: university *Intervention*: 1 day (8 h) group ACT workshop including: creative hopelessness, control as the problem/willingness as the solution, mindfulness and acceptance, values clarification, barriers to values, and committed action. Facilitated by 2 ACT-trained clinical psychologist trainees	Waiting list control condition (completed ACT workshop 2 weeks after intervention group)	Outcome measures: The Physical Appearance State and Trait Anxiety Scale (PASTAS-S) (Reed et al. 1991) The Preoccupation with Eating, Weight and Shape Scale (PEWS; Niemeier et al. 2002); Process measures: Acceptance and Action Questionnaire for Weight (AAQW; Lillis and Hayes, 2008); Acceptance and Action Questionnaire has good reliability and validity (Hayes et al. 2004) *Timing of administration*: Pre- and directly post-intervention, 1 week post intervention, 2 weeks post intervention	*Over time*: Compared to control ACT condition significantly reduced body anxiety with medium effect size (t(58.82) = 2.31, p = 0.025, d = 0.55); reduced preoccupation with eating, weight and shape with large effect size t(105.01) = − 2.71, p = 0.01, d = − 1.40. No significant difference between changes in scores between the ACT and control conditions Effect of ACT condition was significantly mediated by: changes in weight-related psychological flexibility (AAQ-W) with large effect size (F(1, 51.16) = 21.05, p = 0.00, d = 1.28); general psychological flexibility (AAQ) with medium effect size F(1, 60.21) = 6.91, p = 0.01 (d = .68) *Attrition*: 3 of 73 did not attend workshop. 12 out of 34 in control group (35% attrition) and 10 out of 39 ACT group did not complete 1st follow up (26% attrition). 9 out of 34 in control group 26% attritrion and 13 out of 39 (33% attrition) in ACT group did not complete 2nd follow up. Overall 30% attrition
Weineland et al. (2012a); Weineland, et al. (2012b) (6 follow up data)	T: 39, I: 19, C: 20 *Population type*: Participants 6 months post bariatric surgery *Gender*: 35 females and 4 males *Age*: 25–59 years, M = 43.08 (no SD reported) BMI: M = 27.19 (range 20.76—38.01) *Ethnicity*: not reported	*Setting*: local surgery centre and home (via internet) *Intervention* ACT programme for patients who had received bariatric surgery. 8 weekly sessions 2 face-to-face, 6 via Internet, plus 30 min weekly telephone support with trained ACT facilitator Included concepts of acceptance, committed action, values, defusion, and contact with the present moment; acceptance of body image; identified health behaviours values including exercise and eating	*Treatment as usual* participants received follow-up sessions with bariatric surgery team	*Outcome measures*: Body shape questionnaire short version (BSQ) Cooper et al. (1987); Eating disorders examination questionnaire (EDEQ)—weight and shape concern subscales (Fairburn and Beglin 1994) *Process measures*: Acceptance and action questionnaire for weight (AAQ-W; Lillis and Hayes 2008) *Timing of administration*: Pre- and directly post-intervention, 6 months follow up (see Weineland et al. 2012b)	*Post intervention*: Compared to control ACT significantly reduced body shape concerns (BSQ) with medium effect size (F(1,37) = 5.65, p = 0.02, η^2^ = 0.13); significantly reduced shape concerns using EDEQ with large effect (F(1,37) = 7.59, p = 0.009, η2 = 0.17); significantly reduced weight concerns with medium effect (F(1,37) = 5.06, p = 0.03, η^2^ = 0.12) *6 month follow up*: Significant difference in BSQ scores between conditions was maintained with a medium effect size (*t* [58.65] = 2.09, *P* = 0.041, d = 0.77), differences in shape concerns (EDEQ), were maintained with a significant medium effect size (t (59.01) = 1.84, P = 0.07, d = 0.68); no significant difference between conditions for weight concerns was identified Improvements in body dissatisfaction were significantly mediated by improvements in weight-related psychological flexibility (P < 0.05) (point estimate = − 8.16, SE = 4.00, 95% CI: -22.75, -2.67) *Attrition*: 6 of 39 dropped out (15.3%). Out of 3 of 15 (20% attrition) in ACT group and 1 of 18 in control group (5.5% attrition) dropped out at 6 months

*NB T* total number of participants, *I* number of participants in ACT intervention group, *C* number of participants in the control group

All six interventions used an ACT programme based on the original manual by Hayes et al. (1999) and were delivered by ACT-trained therapists (in conjunction with online delivery in Weineland et al. 2012a, b) or a self-help book called “The Diet Trap” (Lillis et al. 2014) in Levin et al’s (2017) intervention. Two studies adopted a treatment as usual group (TAU) as a control (Palmeira et al. 2017a, b; Weineland et al. 2012a), and three studies used waiting list control groups. Levin et al’s (2017) study had no control group.

Studies employed a variety of measures to assess BID or weight self-stigma. Pearson et al. (2012) used the Physical Appearance State and Trait Anxiety Inventory–State Version (PASTAS; Reed et al. 1991) and the Preoccupation with Eating, Weight, and Shape Scale (PEWS; Niemeier et al. 2002). Weineland et al. (2012a) used the weight and shape concerns subscales from the Eating Disorders Examination Questionnaire (EDEQ) and the Body Shape Questionnaire short version (BSQ; Cooper et al. 1987). Lillis et al. (2009) and Fletcher (2011) used the original 30-item Weight Self-Stigma Questionnaire, which was designed by Lillis et al. specifically for their study. Levin et al. (2017) and Palmeira et al. (2017a, b) used the 12-item version of the Weight Self-Stigma Questionnaire (Lillis et al. 2010). Post-intervention outcome measures were administered within different timeframes: Fletcher (2011) administered outcome measures at 1 week and 3 months post intervention; Lillis et al. (2009) repeated assessment at 3 month follow-up; Pearson et al. (2012) included 1- and 2-week follow-ups; Palmeira et al. (2017a, b) followed up within 2 weeks post-intervention and again at 3 months; Levin et al. (2017) followed up directly post-intervention and 3 month follow-up; and Weineland et al. (2012b) followed up directly post-intervention and at 6 months post-intervention.

All but two studies that utilised an RCT design described an appropriate method of randomisation: using computer-based randomisation (Palmeira et al. 2017a, b; Weineland et al. 2012a), coin flip (Pearson et al. 2012) and a random numbers table (Lillis et al. 2009). Levin et al. (2017) did not include a control group and therefore did not conduct randomisation of participants. Fletcher (2011) did not describe the method of randomisation, which resulted in an unclear risk of selection bias, increasing the likelihood of the exaggerated effect sizes. Methods of allocation concealment were not reported in any study and email communication with all authors identified that suitable methods were unlikely to have been used. Overall, lack of allocation concealment suggests high risk of selection bias and magnified reporting of effect sizes.

As is typical in psychological intervention research, blinding of participants was not possible in any study. Similarly, outcome assessors were not blinded to participants’ condition in any study due to the self-reported nature of outcome measures. Palmeira et al. (2017a) blinded the clinical psychologists conducting data collection to the participants’ assigned condition. However, participants were not blinded to the outcome assessment since they self-reported their answers to the questionnaires. Risk of performance and detection bias was therefore considered to be high. Attrition rates were 30% directly post-intervention (Levin et al. 2017), 30% at 2 weeks (Pearson et al. 2012), 7.5, 15 and 18.9% at 3 months (Lillis et al. 2009, Palmeira et al. 2017a, b, and; Fletcher 2011, respectively) and 12% at 6 month follow-up (Weineland et al. 2012b). There were no significant differences in attrition rates between the intervention group and control in all studies that included a control, indicating the studies show an overall low risk of attrition bias.

All studies other than Levin et al., (2017) and Pearson et al. (2012) conducted power analyses. Of the five studies that included a control, Pearson et al. (2012), Palmeira et al. (2017a) and Weineland et al. (2012a) used an intention to treat approach to data analysis, and Lillis et al. (2009) and Fletcher (2011) used a per protocol approach. All studies reported the results of all measures described in their methods, and clearly described how the data was analysed and the number of participants included in the analysis, resulting in a low risk of selective reporting bias. Lillis et al. (2009) reported a significant difference at baseline between the ACT and control group in previous success at losing weight through dieting, but this was adequately addressed by the authors by including previous dieting success as a covariate in the analysis. Otherwise there were no significant differences between group outcome measure scores and other key variables at baseline. Sources of other bias were identified across studies. No studies included an attention control. Whether the benefits of the intervention were due to the ACT techniques specifically or as an effect of receiving an intervention of any kind is therefore unclear. The therapeutic alliance between the ACT facilitator/coach and participants may also have influenced intervention effects.

### Main Findings

Despite adopting a broad inclusion criteria only a small number of eligible studies were identified for review. Duration of the ACT interventions varied (one-day workshops in three studies, a seven-session, eight-session and twelve-session programme in three studies), and a variety of measures targeting different constructs of body image and weight self-stigma were administered. This heterogeneity across studies rendered a meta-analysis unsuitable; a narrative synthesis was therefore applied.

The principle summary measures were the difference in means between the intervention and control groups’ pre and post intervention scores. Effect sizes and p-values were reported. Of the six reviewed studies, analyses of changes in post-treatment outcome measures of BID and weight self-stigma revealed that four studies showed a significant effect of ACT compared to control, with reported Cohen’s d effect sizes ranging from medium to large (*d* = 0.68–2.63, where effect sizes of *d* ≥ 0.2 are deemed small, *d* ≥ 0.5 medium and *d* ≥ 0.8 large, Cohen 1977) and partial eta squared effect sizes ranging from medium to large (η^2^ = 0.12–0.17) (Partial eta square effect sizes: 0.01 = small, 0.06 = medium, 0.14 = large). Levin et al. (2017) did not include a control, however a large significant effect of ACT on weight self-stigma from pre to post intervention was identified. Fletcher (2011) found no significant differences between ACT and control. Taken together, the studies presented methodological issues including small sample sizes, lack of allocation concealment, reliance on self-report, and homogeneity of participants—mainly overweight and obese Caucasian women. These issues prevent conclusions being drawn to a wider population, including other ethnic groups, men and people with a healthy weight.

### The Effect of ACT on BID

Two studies tested the effect of an ACT intervention on direct measures of BID. In the study conducted by Pearson et al. (2012), 73 female participants (aged 18–68, mean age 43.4, SD = 14.7) who responded to an advert and self-reported with BID were randomly assigned to either a one-day (8 h) ACT workshop, or waiting list control plus self-monitoring. The ACT condition resulted in a significant medium reduction in body anxiety compared to control two weeks post-intervention. A significant large reduction in preoccupation with thoughts regarding eating, weight, and shape was also identified in the ACT condition over time. However there was no significant difference in preoccupation with thoughts regarding eating, weight, and shape between the ACT and waitlist control condition in scores over time.

Weineland et al. (2012a) included 39 participants (35 women and 4 men, aged 25–59) who had undergone bariatric surgery at least 6 months previously. Participants were randomly assigned to eight weekly sessions of ACT or to TAU control. Participants in the ACT condition were given two face-to-face sessions in a hospital surgery department and six internet sessions, with a 30 min weekly support session over the telephone. At post-intervention the ACT condition significantly reduced body shape concerns with a medium effect size compared to TAU control. The significant difference in body shape concerns between conditions was maintained at 6 month follow-up, with a medium effect size (Weineland et al. 2012b). The ACT condition had a significant medium effect on reducing post-intervention weight concerns compared to control. However, no significant difference between conditions for weight concerns was identified at 6 months post-intervention. Overall these two studies resulted in medium and large significant improvements BID measures.

### The Effect of ACT on Weight Self-Stigma

Four studies (Lillis et al. 2009; Palmeira et al. 2017a; Levin et al. 2017; Fletcher 2011) investigated the impact of ACT on weight self-stigma in overweight and obese adults. Lillis et al. (2009) recruited 84 participants (76 female, mean age = 51.7) who had completed at least 6 months of any structured weight loss program in the previous 2 years. Participants were randomly assigned to a waiting list control group or an intervention group that received a one-day (6 h) ACT workshop using weight self-stigma as the focus. At 3 months post-intervention the ACT condition had a significant large effect on reducing weight self-stigma compared to control. The ACT group lost significantly more weight than the control. However, further analysis indicated that this weight loss was not responsible for the significant reductions in weight self-stigma.

Fletcher (2011) recruited 72 participants (60 female, mean age = 52.6) who were current or previous patients of an established university laboratory-based structured weight-loss program. Participants were randomly assigned to either a one day (6 h) ACT workshop designed to increase participants’ physical activity, or to a waiting list control. The primary focus of the intervention was on physical activity rather than BID, however the workshop covered the role of weight self-stigma as a barrier to exercise. No significant differences were identified in weight self-stigma between conditions at post-intervention or at 3 month follow-up.

Palmeira et al’s (2017a, b) study recruited 73 overweight or obese women aged 18–55 who reported weight self-stigma. Participants were randomly assigned to either a 12-session face-to-face ACT and self-compassion intervention designed to reduce weight self-stigma, or to a TAU control group. Compared to TAU, the ACT condition had a significant large effect on weight self-stigma post-intervention. In a follow-up study, reductions in weight self-stigma from baseline to post-treatment were maintained after 3 months (Palmeira et al. b). However, data reporting weight self-stigma levels in the control group at 3 month follow-up was not included. The ACT condition resulted in greater weight loss (BMI) compared to control. However, post-hoc analyses showed that improvements in weight related self-stigma were not due to weight loss in the ACT group.

Levin et al. (2017) recruited 13 participants (12 female) aged 18–60 struggling with weight self-stigma with a BMI of 27.5 or above. The intervention involved seven weekly ACT self-guided sessions using an ACT self-help book for weight self-stigma, plus weekly 5–10 min coaching sessions. At 3 month follow-up, the ACT condition showed a significant large effect on weight self-stigma compared to baseline.

All studies that utilised multiple weekly sessions found large significant effects in reducing weight self-stigma (Palmeira et al. 2017a, b; Levin et al. 2017). However, Levin et al’s (2017) findings are limited by not including a control. Studies that used a single day intervention showed mixed results (Fletcher 2011; Lillis et al. 2009). Fletcher (2011) did not show a significant effect of ACT on weight self-stigma, whereas Lillis et al. (2009) found a large significant effect. It should be noted that Fletcher (2011) only dedicated a small part of the protocol to weight self-stigma, whereas Lillis et al’s (2009) one-day ACT protocol specifically targeted weight self-stigma.

### Process Variables Results

Five studies included ACT process outcome measures (i.e. psychometric measures which measure the key ACT processes which are targeted in ACT interventions: acceptance, cognitive defusion, contact with the present moment, self as context, value-driven behavior, and committed action towards value-driven behaviors) to test proposed mediating variables of the interventions’ effects on body image or weight self-stigma (Levin et al. 2017; Lillis et al. 2009, Palmeira et al. b; Pearson et al. 2012; Weineland et al. 2012a) (see Table 1). Fletcher (2011) tested weight self-stigma as a process rather than an outcome variable, as the authors sought to test weight self-stigma as a mediator of increased physical activity (the target of the ACT intervention). Therefore no process variables were introduced to test the intervention’s effect on weight-self stigma in this study. Of the two studies that examined BID, improvements in weight-specific psychological flexibility significantly mediated improvements in body image outcomes (Pearson et al. 2012; Weineland et al. 2012a). Pearson et al. (2012) found that improvements in general ACT processes also mediated improvements in body image.

Three out of the four studies that aimed to reduce weight self-stigma included ACT process measures. Lillis et al. (2009) found that increases in weight-related psychological flexibility and general psychological flexibility mediated the improvements in weight self-stigma in the ACT condition. Palmeira et al. (2017b) found that the significant reduction in weight self-stigma in the ACT conduction was mediated by decreased levels of weight-related psychological inflexibility, shame, self-judgment patterns and increased self-compassion and mindfulness. Levin et al. (2017) found significant reductions in weight-related psychological inflexibility, values obstruction (i.e. how much barriers get in the way of valued action) and increased progress towards valued action in the ACT condition. However, no mediation analysis was conducted between the process variables and weight self-stigma, leaving it unclear as to whether these ACT process variables mediated weight self-stigma (Levin et al. 2017).

## Discussion

Overall the findings suggest that there is a lack of research investigating the effectiveness of ACT for improving body image and weight self-stigma. This review indicates that ACT for BID and weight self-stigma shows promise. However, due to the small number of studies and inconsistent findings, the effectiveness of ACT for BID and weight self-stigma remains unclear. The reviewed studies also presented methodological issues (small sample sizes, lack of allocation concealment, a variety of comparison groups, a lack of attention control and lack of long-term follow up) that impacted the validity of the findings.

### Intervention Duration and Format

Overall the review identified that a variety of intervention durations (multiple weekly sessions vs. a one-day workshop) and formats (face-to-face, online and a self-help book) yielded similar results. All studies apart from Fletcher (2011) reported similar large effect sizes on BID or weight self-stigma, revealing no dose–response relationship. This suggests ACT therapists and researchers can be flexible in how they deliver ACT, and that self-help provided online or via literature can offer an effective resource for geographically remote patients.

### Process Variables

Of the five studies that included process variables, all showed improvements in weight-related ACT processes in the intervention groups, which mediated improvements in BID and weight self-stigma in four studies. The two studies that included measures of general psychological flexibility reported that improvements in general ACT processes also mediated improvements in the body image measures. Another study found that other ACT-related process measures self-judgment patterns, increased self-compassion and mindfulness also mediated the ACT intervention effects. Future ACT studies may consider including both general and weight/appearance related ACT measures to allow for a more thorough investigation of ACT processes on intervention effects.

### Limitations and Future Directions

Across the included studies, allocation concealment was not conducted during randomisation and no attention control was included. To reduce these potential biases, future studies should employ and report adequate allocation sequence concealment and include an attention control condition to enable a more thorough investigation of intervention effects.

A final limitation of the reviewed studies is the homogeneity of participants, who were adult and mainly overweight or obese white women. This prevents generalisations to a wider population. Recommendations for future work therefore include an investigation into the effectiveness of ACT for BID and weight self-stigma in men, children, teenagers and people from ethnic minority groups, those with a condition or injury that affects appearance, and those in ‘normal’ or ‘underweight’ BMI categories. Detailed investigation into the effectiveness of remotely-delivered ACT (e.g. via online methods) and a comparison of the effectiveness of ACT compared to other psychotherapies for BID and weight self-stigma would also be worthy of future investigation. In conclusion, this systematic review highlights that ACT interventions for BID and weight self-stigma shows promise, and investment in future research using larger-scale, higher quality randomized controlled trials is justified and required to confirm the effectiveness of ACT in this area.
